# Recent advances in artificial intelligence-assisted medical imaging education

**DOI:** 10.3389/fmed.2026.1873826

**Published:** 2026-07-02

**Authors:** Zhu Zhu, Tong Zhang, Liping Deng, Yuan Yuan, Hehan Tang, Yi Wei, Qian Li

**Affiliations:** Department of Radiology, West China Hospital, Sichuan University, Chengdu, China

**Keywords:** artificial intelligence, curriculum, education, medical image, radiology

## Abstract

**Introduction:**

The deep integration of artificial intelligence (AI) and medical imaging represents a major trend in the transformation of healthcare, driving advancements in technologies such as image reconstruction. At the same time, medical schools worldwide are integrating AI into medical imaging education. This paper reviews recent advances in artificial intelligent medical imaging education and offers recommendations regarding curriculum design and faculty development for training professionals in artificial intelligent medical imaging.

**Methods:**

This study analyzed the application of AI in medical imaging education and corresponding talent development models through a literature review of core databases such as PubMed and Web of Science, supplemented by case studies, to draw conclusions and propose targeted recommendations.

**Results:**

AI has been widely applied in medical imaging education to enhance educational quality and other aspects. However, globally, AI-related radiology education exhibits inconsistencies in curriculum design and insufficient integration of technology. Although preliminary evidence suggests that AI can effectively improve teaching outcomes, the lack of standardized teaching guidelines has led to gaps in the knowledge system.

**Conclusion:**

The integration of AI and medical imaging offers significant advantages in medical imaging education. However, while the education sector has already adopted various strategies—such as human-machine collaborative education—it still faces challenges, including a shortage of interdisciplinary faculty and a disconnect between the curriculum and clinical practice. Improvements must be made through strategies such as faculty development, pedagogical transformation, fostering AI literacy, and standardizing teaching frameworks. Future research should explore the adaptability of AI across different training stages to promote the sustainable integration of these two fields and the development of relevant professionals.

## 1 Introduction

Medical imaging is a cornerstone of modern medicine, providing essential structural and functional information for disease detection, diagnosis, treatment planning, and therapeutic monitoring. Over the past decades, imaging technologies have evolved from conventional radiography to advanced modalities such as computed tomography (CT), magnetic resonance imaging (MRI), and multimodal imaging systems, significantly enhancing diagnostic precision and clinical decision-making (1).

Concurrently, the rapid digitalization of healthcare has led to an exponential growth in imaging data, both in volume and complexity. Traditional radiological workflows, which rely heavily on manual interpretation, are increasingly challenged by high data throughput, time constraints, and inter-observer variability (2). These limitations have created a critical need for more efficient, standardized, and scalable approaches to image analysis.

Artificial intelligence (AI), particularly machine learning and deep learning, has emerged as a transformative force in medical imaging (2). Recent advances have enabled substantial improvements in image reconstruction, noise reduction, lesion detection and segmentation, quantitative analysis, and computer-assisted diagnosis (3). Beyond isolated tasks, AI is increasingly being integrated into end-to-end clinical workflows, reshaping how imaging data are processed, interpreted, and utilized in clinical decision-making. This transformation is not only technological but also conceptual, shifting radiology toward a paradigm of human–AI collaboration.

However, while the technological capabilities of AI in medical imaging have advanced rapidly, a corresponding transformation in medical imaging education has not yet been fully realized. This has led to a growing “competency gap” between the skills required in AI-augmented clinical environments and those currently emphasized in traditional training programs (4). Medical imaging education faces additional challenges, including the complexity of anatomical and multimodal data, limited opportunities for hands-on training, and shortages of faculty with interdisciplinary expertise in both imaging and AI (5).

The integration of AI into medical imaging education offers both opportunities and challenges. On one hand, AI-driven educational tools—such as intelligent tutoring systems, automated image annotation, and adaptive learning platforms—can enhance learning efficiency, support personalized training, and facilitate interactive image interpretation (6–8). On the other hand, the increasing reliance on AI systems raises concerns regarding overdependence, reduced development of independent diagnostic reasoning, and the need to incorporate ethical, regulatory, and interpretability considerations into training programs (2, 4).

Importantly, the impact of AI on education extends beyond the adoption of new tools. It necessitates a fundamental rethinking of educational goals, shifting from knowledge-based training toward competency-based frameworks that emphasize data literacy, algorithmic understanding, and human–AI collaboration (2–4). This transition requires systematic curriculum redesign, interdisciplinary integration, and the development of new assessment strategies aligned with evolving clinical practice.

In this context, the present review aims to provide a comprehensive synthesis of recent advances in AI-assisted medical imaging education. We propose a conceptual linkage between technological innovation and educational transformation, and we analyze current educational practices across different training stages, including undergraduate, postgraduate, and residency levels. Furthermore, we discuss emerging educational strategies, interdisciplinary training models, learner acceptance, and the key challenges that must be addressed to achieve effective and sustainable integration of AI into medical imaging curricula.

## 2 Methods

This study was conducted as a narrative review to summarize recent advances in artificial intelligence (AI)-assisted medical imaging education and to analyze corresponding educational strategies.

### 2.1 Literature search strategy

A comprehensive literature search was performed in major domestic and international databases, including China National Knowledge Infrastructure (CNKI), PubMed, and Web of Science. The search covered studies published up to January 2026.

The search strategy combined keywords related to artificial intelligence (“artificial intelligence,” “machine learning,” “deep learning”), medical imaging (“medical imaging,” “radiology,” “CT,” “MRI”), and education (“medical education,” “radiology education,” “curriculum,” “training”). These terms were used individually and in combination to maximize retrieval.

### 2.2 Inclusion and exclusion criteria

Studies were included if they met at least one of the following criteria: (1) reported recent advances in AI applications within medical imaging education, including but not limited to image reconstruction, segmentation, and diagnostic support; or (2) addressed educational strategies, curriculum development, or training models related to AI in medical imaging education.

Eligible publications included original research articles, review articles, and relevant perspective or policy papers. Studies not directly related to medical imaging education were excluded.

### 2.3 Study selection

Two reviewers independently screened the literature in two stages. First, titles and abstracts were assessed for relevance based on the predefined inclusion criteria. Subsequently, full-text articles were evaluated to confirm eligibility. Any discrepancies between reviewers were resolved through discussion until consensus was reached.

### 2.4 Data extraction and synthesis

Relevant data were extracted and synthesized using a thematic framework. The extracted information was categorized into two main domains: (1) educational statues aspects, including AI imaging education modalities, AI imaging education types, and methodological approaches; and (2) educational strategy aspects, including teaching strategies, evaluation methods, implementation challenges, and future directions.

The synthesized findings were used to structure the subsequent sections of this review, enabling an integrated analysis of the advances of AI-assisted medical imaging education.

## 3 Brief overview of advances in AI-assisted medical imaging

Recent advances in artificial intelligence (AI), particularly deep learning, have substantially expanded the capabilities of medical imaging. By enabling automated feature extraction from large-scale datasets, AI has improved performance across multiple stages of the imaging workflow, including image acquisition, reconstruction, analysis, and clinical decision support (9). These developments are progressively reshaping radiological practice and enhancing both efficiency and diagnostic accuracy.

### 3.1 Advances in AI-assisted CT imaging

In CT, AI applications can be broadly categorized according to their functional roles, including image acquisition and reconstruction, image enhancement, tumor segmentation and diagnostic detection, and prognostic evaluation (10, 11).

AI-based reconstruction and enhancement techniques have demonstrated significant advantages over traditional methods. Deep learning reconstruction (DLR) algorithms can effectively reduce noise, suppress artifacts, faster image reconstruction, reducing scan repetition, and improve spatial resolution, enabling high-quality imaging at reduced radiation doses (10). For example, recent studies have shown that AI models can generate contrast-enhanced images from non-contrast CT scans, thereby reducing the need for repeated imaging and minimizing patient exposure to radiation and contrast agents (12).

A variety of commercially available DLR systems, such as AiCE, TrueFidelity, Precise Image, and ClariCT.AI, have been increasingly implemented in clinical practice (13). In addition, multiple deep learning architectures—including convolutional neural networks (CNNs), generative adversarial networks (GANs), variational autoencoders (VAEs), and residual networks (ResNets)—have been widely applied for CT image denoising and quality enhancement (14). Artificial intelligence optimizes adaptive imaging protocols tailored to individual patient characteristics by intelligently and dynamically adjusting scan parameters, radiation dose, and contrast agent injection timing during the imaging process (15). These advances highlight the growing role of AI in supporting low-dose, high-quality CT imaging and in promoting more standardized and efficient imaging practice.

Beyond image quality improvement and workflow optimization, AI also contributes to assist tumor segmentation, improve diagnostic efficiency and promoting prognostic evaluation (16–19). For instance, AI-based gastric cancer risk assessment program (GRAPE) that utilizes non-contrast CT and deep learning techniques to identify gastric cancer with excellent identification capabilities (area under the curve = 0.927). Significantly outperformed radiologists, with a 21.8% increase in sensitivity and a 14.0% increase in specificity (17);thereby opening up new possibilities for AI to drive clinical decision-making and patient survival rate improvement across assisting the entire lesion diagnosis and treatment pathway.

### 3.2 Advances in AI-assisted MRI

Artificial intelligence has also been extensively applied in magnetic resonance imaging (MRI), particularly in oncological imaging and functional assessment. AI-driven approaches have improved lesion detection, segmentation, and quantitative analysis, thereby enhancing the diagnostic value of MRI (20).

Compared to CT, MRI has the advantage of eliminating concerns about radiation exposure. However, MRI has one major limitation: the lengthy scan time, which makes it prone to motion artifacts, thereby compromising image quality. AI-based MRI reconstruction techniques can shorten scan times while maintaining sensitivity, thereby preventing the occurrence of large-scale motion artifacts (15, 21). We believe that deep learning-based MRI reconstruction techniques can significantly reduce scan times and, following further prospective validation, are expected to substantially improve access to MRI.

AI models have demonstrated strong performance in lesion detection, segmentation and prognostic assessment. Deep learning-based automatic segmentation techniques have demonstrated excellent performance in MRI, enabling accurate quantitative assessment of organ and lesion volumes and providing precise support for clinical diagnosis and treatment. Studies have shown that AI-assisted analysis can improve consistency in lesion detection and, in some cases, achieve performance comparable to that of experienced radiologists (22, 23). Segmentation models are also widely used for organ delineation and treatment planning, including MRI-guided radiotherapy (24). In addition, detection models can facilitate longitudinal lesion monitoring and disease progression assessment (25). Despite these advances, challenges remain in terms of generalizability, standardization, and clinical validation. Nevertheless, current evidence suggests that AI will continue to play an increasingly important role in enhancing MRI-based diagnosis and supporting precision medicine.

## 4 Current status of AI integration in medical imaging education

Medical imaging is a core discipline in modern clinical practice, and the rapid development of artificial intelligence (AI) is increasingly influencing its educational paradigms. In recent years, multiple international organizations have emphasized the importance of incorporating AI into medical imaging training. For instance, the American Society of Radiologic Technologists (ASRT) recommended in its 2024 consensus report that AI should be included as a required component of imaging curricula rather than an elective (13, 26). Similarly, the establishment of dedicated academic platforms, such as the journal *Radiology: Artificial Intelligence*, reflects the growing academic and educational relevance of AI in the field (4). Collectively, these developments indicate a progressive shift toward the integration of AI into medical imaging education.

### 4.1 Integration across different training stages

The adoption of AI in medical imaging education varies across different stages of training, reflecting differences in learning objectives and competency requirements.

At the undergraduate level, AI education is typically introductory, focusing on fundamental concepts, basic applications, and ethical considerations. AI is primarily used as a supplementary teaching tool to support foundational learning (4). AI-assisted systems and online learning platforms can help students further master foundational knowledge—such as anatomy—and enhance their professional skills. In addition, AI applications in medical imaging are already in use. Students should be exposed to these applications and explore them further, as this helps undergraduates develop clinical reasoning and broaden their clinical perspectives.

At the postgraduate level, AI-related education becomes more structured and in-depth, with dedicated courses addressing algorithmic principles, clinical applications, and research methodologies. Programs such as the “Artificial Intelligence in Medical Imaging” course at the University of Sydney and structured educational interventions reported in recent studies demonstrate an increasing emphasis on integrating AI into advanced training (4, 27).

In residency training, AI applications extend beyond theoretical instruction to practical and clinically oriented use (28). Educational programs, such as the RADS curriculum, are designed to help trainees understand both the capabilities and limitations of machine learning and to critically evaluate AI-related literature (29). In addition, AI systems are increasingly used to support personalized training, for example by identifying diagnostic blind spots and tailoring case-based learning accordingly (30).

### 4.2 Learner perceptions and acceptance of AI

Despite the expanding role of AI in medical imaging education, student perceptions remain heterogeneous. Some medical students continue to express concerns regarding the potential impact of AI on the future role of radiologists (3). Recent evidence by Jasper van Hoek et al. (2) has shown that a minority of students may reconsider radiology as a career due to such concerns. They conducted an online survey via the SurveyMonkey platform targeting 170 radiology professionals and students in the German-speaking part of Switzerland., the results showed that among students who did not intend to specialize in radiology, 26% cited AI as one of the reasons.

However, emerging evidence suggests that structured exposure to AI education can positively influence student attitudes (2, 31). For example, students who receive AI-related training are more likely to consider radiology as a career compared to those without such exposure. Furthermore, students with stronger technological backgrounds tend to demonstrate lower levels of concern regarding AI-related disruption (2, 3). Only 15.2% (40 out of 263) of medical students believe that radiologists will be completely replaced by artificial intelligence (3). These findings highlight the importance of early and structured integration of AI into medical education.

### 4.3 Implementation approaches in AI-assisted teaching

Current approaches to integrating AI into medical imaging education primarily include curriculum-based instruction, AI-supported learning platforms, and interdisciplinary training models.

Curriculum integration typically involves the inclusion of foundational AI concepts, such as machine learning and deep learning, as well as their applications in medical imaging (4). These components help students build a conceptual understanding of AI and its clinical relevance.

AI-supported learning platforms enable interactive and adaptive education. These systems can provide automated image annotation, real-time feedback, and performance tracking, thereby improving learning efficiency and supporting individualized training (6–8, 32). For example, AI-assisted diagnostic systems have been shown to improve students’ performance in image interpretation tasks (8). In this regard, a study by Xiaohong Lyu et al. (8) demonstrated that AI-assisted diagnostic systems are an effective tool for training junior radiology residents and medical imaging students in disease detection and diagnosis. The researchers divided the students into three groups and applied different teaching models to each. Using pathological results and radiologists’ diagnostic opinions as the gold standard, they compared the performance of three groups in terms of detection rate, diagnostic agreement, number of false positives per case, and Kappa coefficient across 1,057 tests involving the localization and grading of pulmonary nodules. The results showed that the group using the AI-assisted diagnostic system teaching model demonstrated a greater improvement in test scores and superior diagnostic performance. This further demonstrates that the AI-assisted diagnostic system is an effective tool for training junior radiology residents and medical imaging students in the detection and diagnosis of pulmonary nodules (8).

In addition, generative AI tools have demonstrated potential in supporting assessment design, with evidence indicating that AI-generated examination items can achieve comparable quality to those developed by human instructors (33). This offers a new approach to addressing the challenge of reducing teachers’ workload while maintaining educational quality. A quantitative study by Emre Emekli et al. (33) explored this issue. The researchers asked participants to answer multiple-choice exam questions generated by ChatGPT-4o and by instructors, respectively, and to provide feedback using a 5-point Likert scale. The results showed that the average number of correct answers on the ChatGPT-generated exams was comparable to that on the exams created by human experts (*p* = 0.089). There was also a moderate positive correlation between the scores of the two exams (*r* = 0.628, *p* < 0.001). ChatGPT’s average difficulty index was comparable to that of human experts, and its discrimination index fell within an acceptable range (73.33%, 86.67%). Although some limitations remain regarding discrimination and difficulty alignment, the quality of questions generated by large language models (LLMs) such as ChatGPT is comparable to those created by human experts. These models are expected to provide a valuable supplement to the assessment process in health education.

Interdisciplinary training models, including collaborations between medical and engineering disciplines, further enhance the integration of AI into education. These approaches promote a deeper understanding of algorithmic principles and foster innovation-oriented thinking. Examples include interprofessional education programs and joint research platforms that facilitate collaboration between clinicians and data scientists (34, 35).

### 4.4 Educational value and emerging trends

The integration of AI into medical imaging education provides opportunities to address several limitations of traditional teaching models. AI-assisted approaches can improve learning efficiency, support personalized training, and enhance students’ diagnostic reasoning through interactive and data-driven learning environments (7).

More importantly, the role of AI in education extends beyond the introduction of new tools. It reflects a broader shift toward competency-based training, where emphasis is placed on human–AI collaboration, critical evaluation of algorithmic outputs, and the integration of multimodal information (6, 7, 36). These emerging trends underscore the need for systematic curriculum redesign to align medical imaging education with the evolving demands of AI-augmented clinical practice.

## 5 Educational strategies for AI integration in medical imaging education

In response to the growing integration of artificial intelligence (AI) into clinical imaging practice, medical education has begun to adopt a range of strategies aimed at aligning training with emerging competency requirements. These strategies extend beyond the incorporation of new technologies and reflect a broader shift toward learner-centered, data-driven, and competency-based educational models.

### 5.1 AI-supported intelligent and interactive learning

AI-enabled intelligent learning systems have introduced new opportunities for interactive and adaptive education in medical imaging. These systems can integrate domain-specific knowledge and provide real-time assistance in image interpretation, automated feedback, and performance tracking, thereby enabling precise education in the field of medical imaging (3, 6, 7, 36).

Through interactive learning environments, students can engage in iterative practice by analyzing imaging cases, receiving immediate feedback, and refining their diagnostic reasoning (36). AI-based platforms can also monitor learners’ progress, identify knowledge gaps, and dynamically adjust learning pathways to support individualized training (3, 8). Students can also gain an in-depth understanding of artificial intelligence and data science (4). Such approaches have been shown to improve learning efficiency and enhance students’ engagement with complex imaging tasks.

Importantly, AI-supported learning also facilitates the development of critical thinking by encouraging students to evaluate and interpret AI-generated outputs rather than passively accepting them (3, 36, 37).

### 5.2 Human–AI collaborative teaching models

Given the interdisciplinary nature of medical imaging, collaborative teaching models integrating students, educators, and AI systems have gained increasing attention (5). In these models, AI functions as an auxiliary instructional agent, serve as a “smart mentor” supporting in the process of teaching and learning (3, 5).

While educators remain responsible for curriculum design and knowledge delivery, AI systems can assist in managing learning activities, analyzing student performance, and providing personalized guidance (3). This tripartite interaction establishes a feedback loop among students, teachers, and AI, enabling continuous adaptation of teaching strategies based on learner needs (38).

Such human–AI collaborative frameworks represent a shift from traditional teacher-centered instruction toward more flexible and responsive educational environments.

### 5.3 Virtual simulation and immersive learning

Virtual simulation technologies, including virtual reality (VR) and AI-assisted simulation platforms, have become increasingly important in medical imaging education (3). These approaches enable learners to perform imaging procedures and analyze cases within controlled virtual environments (5).

By transforming two-dimensional imaging data into three-dimensional and interactive representations, virtual simulation enhances spatial understanding and facilitates the comprehension of complex anatomical structures (6). In addition, simulation-based learning allows students to manipulate imaging parameters and observe corresponding changes, thereby strengthening their conceptual understanding and increasing diagnostic confidence (5, 32).

AI-powered virtual teaching assistants can further support simulation-based education by providing real-time guidance and answering student queries, reducing instructional burden and improving learning efficiency (3).

### 5.4 Online and resource-based learning

Online education platforms have become an essential component of medical imaging education, particularly in supporting self-directed learning. AI technologies can enhance these platforms by enabling personalized content recommendations, intelligent search functions, and adaptive learning pathways.

Students can access diverse educational resources, including clinical cases, lectures, and question banks, through digital platforms such as MOOCs and specialized medical databases (such as MedlinePlus and UpToDate) (39). AI systems can further support learning by organizing content, generating summaries, and providing targeted review materials (33).

While these approaches improve accessibility and resource distribution, variability in content quality remains a significant concern, highlighting the need for standardized educational frameworks and quality control mechanisms.

### 5.5 AI-assisted assessment and feedback

Assessment is a critical component of medical imaging education, particularly in competency-based training models (4, 37). AI has been increasingly applied to both summative and formative assessment processes.

AI-driven assessment systems can generate examination items, analyze student performance, and provide personalized feedback. These systems enhance the efficiency and objectivity of evaluation while supporting individualized learning. In addition to formal examinations, emerging AI tools such as Khamingo, AI can facilitate continuous formative assessment by analyzing students’ responses and identifying areas of misunderstanding (3, 36, 37).

Moreover, educational approaches that involve critical evaluation of AI-generated content—such as peer review and reflective analysis—can help reduce overreliance on AI systems and strengthen learners’ practical skills for identifying and evaluating potential biases (4).

Overall, AI-assisted assessment contributes to a more dynamic and feedback-oriented learning environment, supporting both knowledge acquisition and competency development (Table 1).

**TABLE 1:** Educational strategies for AI integration in medical imaging education.

Strategy	Description	Benefits
AI-supported intelligent and interactive learning	The AI-powered learning system integrates knowledge from the field of medical imaging to provide image interpretation, real-time feedback, and progress tracking. It creates an interactive environment that dynamically adjusts learning paths, guiding students to evaluate AI results rather than passively accept them.	Boosts learning efficiency and engagement in complex imaging tasks. Deepens AI/data science understanding and fosters critical thinking.
Human–AI collaborative teaching models	AI serves as an “intelligent tutor,” forming a three-way collaboration with teachers and students. AI assists with instructional management and personalized guidance, while teachers lead course design and knowledge delivery.	Shifts to flexible, responsive education from teacher-centered instruction. Optimizes teaching resource allocation and reduces educator burden.
Virtual simulation and immersive learning	Using VR and AI simulation platforms to perform imaging procedures, analyze medical records, and interact with 3D anatomical models in a virtual environment.	Enhances spatial understanding and diagnostic confidence. Improves learning efficiency and lessens educator instructional load.
Online and resource-based learning	Leveraging online platforms such as MOOCs and specialized databases, AI provides personalized recommendations, intelligent search, and adaptive learning paths.	Supports self-directed learning and resource accessibility. Facilitates targeted review and knowledge consolidation
AI-assisted assessment and feedback	AI is used for formative and summative assessments to automatically generate questions, analyze responses, identify areas for improvement, and provide personalized feedback.	Improves evaluation efficiency and objectivity. Reduces AI overreliance and builds practical diagnostic skills. Creates a dynamic, feedback-driven learning environment.

## 6 Challenges in AI-integrated medical imaging education

Despite the rapid development of artificial intelligence (AI) in medical imaging and its increasing incorporation into educational practice, several challenges remain that hinder effective and sustainable integration. These challenges span multiple domains, including faculty expertise, curriculum standardization, resource availability, alignment with clinical practice, and the reliability of AI systems.

### 6.1 Variability in teaching quality and faculty expertise

One of the most prominent challenges is the heterogeneity in teaching quality across institutions, largely driven by differences in faculty expertise (40, 41). The interdisciplinary nature of AI-assisted medical imaging requires educators to possess both domain-specific imaging knowledge and a foundational understanding of AI methodologies. Research indicates that although most Magnetic Resonance Tomography (MRT) educators are familiar with the basic concepts and definitions of artificial intelligence, 49% said they had not received adequate training to learn and apply new technologies, including artificial intelligence, machine learning, and automation (4, 13, 42). However, such expertise remains unevenly distributed, particularly across regions with differing levels of educational and healthcare resources (2).

Limited faculty expertise may restrict students’ access to high-quality and up-to-date educational content and may also hinder the effective integration of interdisciplinary perspectives. This issue underscores the need for structured faculty development programs and interdisciplinary teaching models to support the delivery of AI-related content.

### 6.2 Lack of standardized curriculum frameworks

The absence of standardized curricula represents another critical barrier (5). Current educational content varies substantially across institutions due to differences in textbook selection, curriculum design, and instructional priorities (4). As a result, students may develop inconsistent levels of knowledge and clinical competence.

In the absence of unified guidelines, educators often rely on personal experience or institutional preferences when designing AI-related content, leading to fragmentation in training outcomes (43). The development of competency-based, standardized curriculum frameworks is therefore essential to ensure consistency and comparability in medical imaging education (13, 42).

### 6.3 Limited access to educational and technical resources

Resource constraints remain a significant limitation (26), particularly in institutions with limited access to advanced imaging equipment and clinical case databases (42). The high cost of imaging systems and the restricted availability of real-world clinical data limit opportunities for hands-on training.

Consequently, students may rely heavily on static or low-quality educational materials, which may not adequately reflect current clinical practice. In addition, limited exposure to advanced imaging technologies may hinder the development of spatial reasoning and practical skills that are critical for clinical competence (44).

### 6.4 Misalignment between educational content and clinical practice

The rapid evolution of imaging technologies and AI applications often outpaces curriculum updates, resulting in a disconnect between educational content and current clinical practice (41). Outdated teaching materials may fail to incorporate recent advances in AI, limiting students’ preparedness for real-world clinical environments (5, 45).

Furthermore, uncertainty among educators regarding the relevance and depth of AI-related content may contribute to inconsistent curriculum implementation (46). Addressing this gap requires continuous curriculum revision and stronger integration among educational institutions, regulatory bodies, and clinical practice settings (4).

### 6.5 Variability and reliability of AI systems

The variability in performance and reliability of AI systems presents an additional challenge in educational settings. Artificial intelligence systems are unpredictable, and students should be required to consider various dilemmas (4). AI-generated outputs may vary across platforms and may occasionally contain inaccuracies, inconsistencies, or insufficient depth. Such variability can affect learning quality and may lead to misconceptions if not critically evaluated.

Moreover, the uneven distribution of AI capabilities across different knowledge domains may result in fragmented learning experiences. These challenges highlight the importance of incorporating critical appraisal skills into training and ensuring that AI tools are used as supportive, rather than authoritative, educational resources (47).

## 7 Future directions in AI-integrated medical imaging education

As artificial intelligence (AI) continues to reshape medical imaging practice, future educational development must move beyond incremental adjustments toward systematic and evidence-based transformation. Several key directions can be identified to support the sustainable integration of AI into medical imaging education.

### 7.1 Faculty development and pedagogical transformation

Future efforts should prioritize the development of faculty expertise in both medical imaging and AI (48). The effective integration of AI into education depends not only on technological tools but also on educators’ ability to interpret, contextualize, and teach these technologies (41).

Faculty development programs should therefore focus on interdisciplinary training, combining imaging knowledge with foundational AI literacy (48). In addition, pedagogical approaches must evolve from traditional knowledge transmission toward facilitation of critical thinking, problem-solving, and human–AI collaborative skills (4, 13, 42). Future research is needed to evaluate the effectiveness of different faculty training models and their impact on learning outcomes.

### 7.2 Development of AI literacy and competency-based training

A central direction for future education is the establishment of competency-based frameworks that define the core skills required in AI-augmented imaging practice. These competencies may include data literacy, understanding of algorithmic principles, critical appraisal of AI outputs, and the ability to integrate AI into clinical decision-making (49). We agree with studies from Canada and Germany that medical school curricula should address artificial intelligence and its potential impact on radiology (2).

Educational strategies should also focus on cultivating students’ AI literacy, enabling them to understand the capabilities and limitations of AI systems and to use them appropriately (49). A report on postgraduate education in the United Kingdom notes that educational strategies should incorporate concepts related to artificial intelligence and data science, ethical considerations regarding the implementation of artificial intelligence in various forms of radiography, the use of artificial intelligence tools in medical imaging, and frameworks for the evaluation and validation of artificial intelligence (2, 4). Future research should aim to identify optimal methods for teaching AI concepts to learners with diverse backgrounds and to assess long-term impacts on clinical competence (50, 51).

### 7.3 Standardization of curriculum and educational frameworks

The development of standardized curricula represents a critical step toward reducing variability in training and ensuring consistent educational outcomes. Educational programs should be continuously updated to reflect the guidelines issued by the governing body (4). Future work should focus on defining core learning objectives, curriculum structures, and assessment criteria for AI-integrated medical imaging education, including basic computational skills, advanced image processing techniques, and state-of-the-art AI tools, to enhance the quality of education in multiple intelligences and prepare students for their future development (47).

Such frameworks should be adaptable to different educational contexts while maintaining consistency in competency requirements. In addition, evidence-based curriculum design and continuous updating mechanisms are needed to ensure alignment with rapidly evolving clinical technologies (5, 52).

### 7.4 Integration of digital and simulation-based learning environments

Advances in digital technologies, including virtual simulation and AI-enhanced learning platforms, are expected to play an increasingly important role in future education. These tools can support immersive learning, provide scalable training solutions, and enable individualized learning experiences (6, 53).

Future research should investigate the effectiveness of simulation-based and AI-supported learning environments in improving diagnostic accuracy, spatial reasoning, and clinical decision-making (51). In addition, the integration of these technologies into formal curricula requires careful evaluation to ensure educational value and sustainability (54).

### 7.5 Resource allocation and educational equity

Ensuring equitable access to high-quality educational resources remains a key challenge for future development (41, 42). Differences in institutional resources, access to imaging equipment, economic costs, and availability of AI tools may contribute to disparities in educational outcomes (49, 55).

Future strategies should focus on optimizing resource allocation, promoting shared educational platforms, and supporting international collaboration to reduce regional inequalities. Research is also needed to evaluate the impact of resource distribution on training quality and learner performance.

### 7.6 Governance, ethics, and responsible use of AI

As AI becomes increasingly embedded in education, issues related to governance, ethics, and responsible use must be addressed (2, 4). According to the European Society of Radiology and a study on the ethics of radiographers, future educational strategies should include the following topics: preventing academic misconduct, ensuring transparency of AI-generated content, and promoting responsible use of AI tools (4).

Future educational frameworks should incorporate ethical training and regulatory awareness as core components. In addition, further research is required to develop guidelines for the safe and effective use of AI in educational settings (54).

**FIGURE 1:**
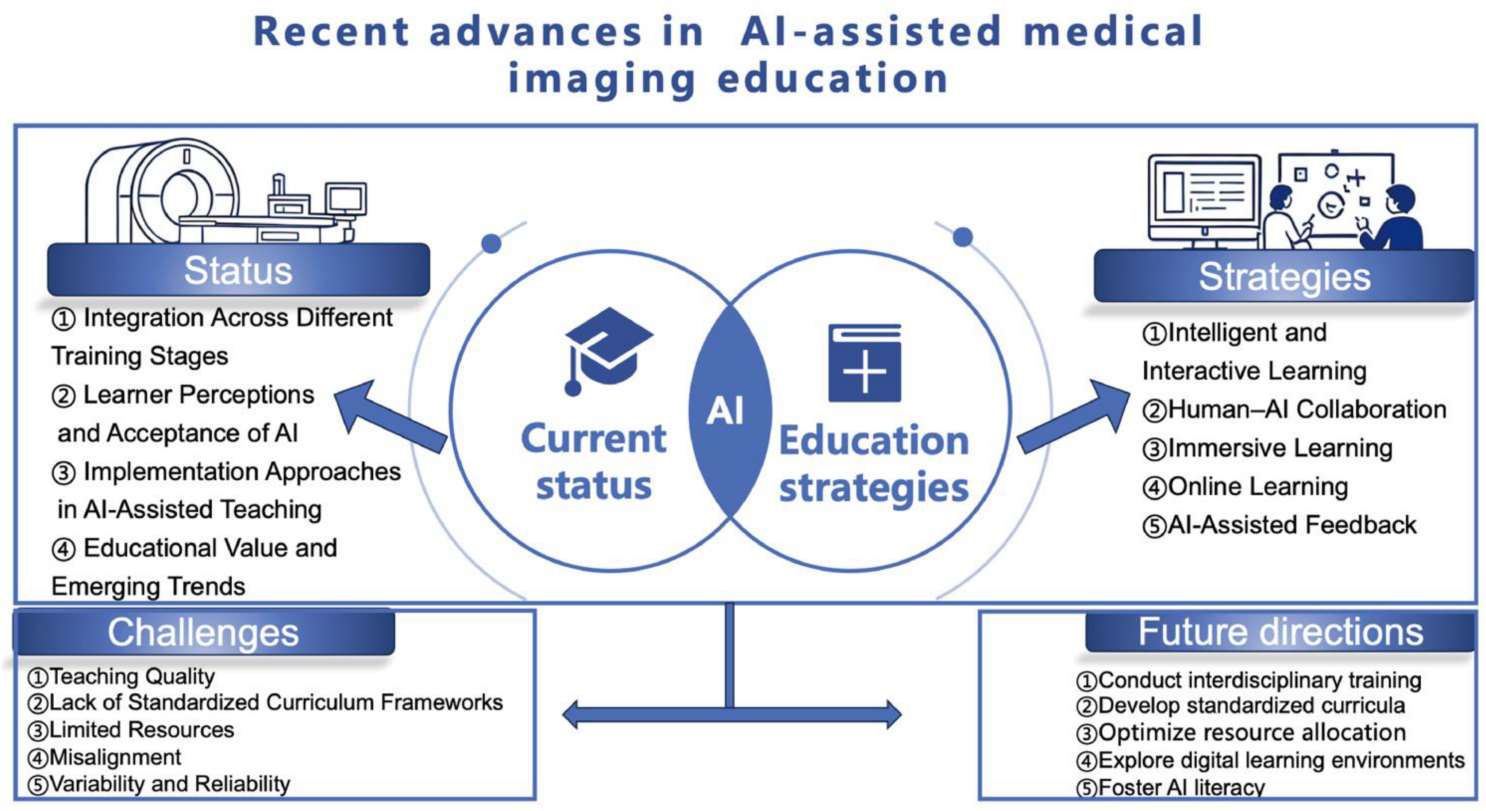
The summary chart of recent advances in AI-assisted medical imaging education.

Overall, future directions in AI-assisted medical imaging education should focus on aligning technological innovation with competency-based training, evidence-based curriculum design, and responsible implementation, thereby ensuring that educational systems are prepared for the evolving demands of AI-enabled clinical practice (49, 53, 54; Figure 1 and Table 2).

**TABLE 2:** Challenges and future directions in AI-integrated medical imaging education.

Challenge	Description	Impact on education	Future directions
Variability in teaching quality and faculty expertise	Faculty expertise in AI and medical imaging is unevenly distributed and 49% of MRT educators lack adequate training in AI/ML/automation.	Restricts students’ access to high-quality, up-to-date content and hinders interdisciplinary teaching integration	Enhance teachers’ dual-discipline competencies, conduct interdisciplinary training, and transform teaching methods
Lack of standardized curriculum frameworks	Courses vary widely, lack standardized guidance, and training is fragmented.	There is a disparity between students’ knowledge levels and clinical skills, and their achievements are fragmented.	Develop standardized curricula, define core objectives and assessment criteria, and establish a mechanism for updates
Limited access to educational and technical resources	Some institutions have limited resources and face difficulties in obtaining advanced equipment and data.	This limits opportunities for hands-on training and hinders the development of spatial reasoning skills and practical abilities.	Optimize resource allocation, promote shared platforms, and strengthen international cooperation.
Misalignment between educational content and clinical practice	The curriculum is out of step with clinical practice, and the materials are outdated.	Outdated materials limit students’ clinical preparedness.	Exploring the educational effectiveness of blended learning environments that integrate digital and analog elements
Variability and reliability of AI systems	AI systems vary in performance, and their outputs may occasionally be inaccurate.	Impairs learning quality and may cause misconceptions and leads to fragmented learning experiences.	Fostering students’ AI literacy, integrating ethics and governance, and regulating the use of AI.

## 8 Conclusion

Artificial intelligence (AI) has substantially advanced medical imaging and is progressively reshaping both clinical practice and educational paradigms. As AI technologies become increasingly integrated into imaging workflows, the competencies required of future imaging professionals are undergoing a corresponding transformation.

Current evidence indicates that AI-assisted educational approaches have the potential to enhance learning efficiency, support personalized training, and facilitate the development of diagnostic and analytical skills. However, several challenges remain, including variability in teaching quality, lack of standardized curricula, limited resource availability, and concerns regarding the reliability and appropriate use of AI systems.

Addressing these challenges requires coordinated efforts in faculty development, competency-based curriculum design, resource optimization, and the establishment of clear educational frameworks. Importantly, the integration of AI into medical imaging education should not be viewed solely as the adoption of new technologies, but as a broader transformation toward human–AI collaborative learning and evidence-based educational practice.

Future research should focus on evaluating the effectiveness of AI-supported educational strategies, developing standardized training models, and ensuring the responsible and equitable implementation of AI in educational settings. Such efforts will be essential to align medical imaging education with the evolving demands of AI-enabled clinical practice.
